# Preparation and Characterization of New pH-Sensitive Polyurethane Hydrogels as Anti-Cancer Drug Delivery Systems for 5-Fluorouracyl and Fluorodeoxyuridine

**DOI:** 10.3390/ijms262110258

**Published:** 2025-10-22

**Authors:** Marcin Sobczak, Adam Kasiński, Karolina Kędra, Joachim Frankowski, Matylda Kurzątkowska, Karolina Watrakiewicz, Karolina Mulas, Katarzyna Strzelecka, Marcin Chodkowski, Małgorzata Krzyżowska, Andrzej Deptała, Ewa Oledzka

**Affiliations:** 1 Department of Pharmaceutical Chemistry and Biomaterials, Faculty of Pharmacy, Medical University of Warsaw, Banacha 1 Str., 02-097 Warsaw, Poland; adam.kasinski@wum.edu.pl (A.K.); joachim.frankowski@wum.edu.pl (J.F.); karolka2804@interia.pl (K.W.); karolina.mulas@wum.edu.pl (K.M.); katarzyna.strzelecka@wum.edu.pl (K.S.); eoledzka@wum.edu.pl (E.O.); 2 Institute of Physical Chemistry, Polish Academy of Sciences, Kasprzaka 44/52 Str., 01-224 Warsaw, Poland; kkedra@ichf.edu.pl; 3 Division of Medical and Environmental Microbiology, Military Institute of Hygiene and Epidemiology, 01-063 Warsaw, Poland; marcin.chodkowski@wihe.pl (M.C.); malgorzata.krzyzowska@wihe.pl (M.K.); 4 Department of Oncology, National Medical Institute of the Ministry of the Interior and Administration, 02-507 Warsaw, Poland; andrzej.deptala@wum.edu.pl; 5 Department of Oncology Propaedeutics, Medical University of Warsaw, 01-445 Warsaw, Poland

**Keywords:** drug delivery systems, biomedical hydrogel, anti-cancer drug, biomedical polyurethane, biomedical polyesters, controlled anti-cancer drug release

## Abstract

In this study, non-toxic, biodegradable, and pH-sensitive polyurethane hydrogels (PUs) were prepared by using hexamethylene diisocyanate (HDI), copolymers of є-caprolactone (CL), *rac*-lactide (LA), and poly(ethylene glycol) (PEG), poly(ethylene glycol)-*block*-poly(propylene glycol)-*block*-poly(ethylene glycol) (PEO-bPPO-b-PEO), 1,4-butanediol (BD), and L-glutamine (Gln). The CL, LA, and PEG copolymers were obtained in the presence of a new synthesized catalytic system: diethylzinc/ethyl-3,4-dihydroxybenzoate. Obtained PUs were screened for their cytotoxicity, evaluated for their swelling behavior and hydrolytic degradation, and employed as hydrogel pH-responsive anti-cancer drug delivery systems (DDSs). The novel and promising hydrogel DDSs, capable of releasing 5-fluorouracyl (5-FU) and fluorodeoxyuridine (5-fluoro-2′-deoxyuridine, FUdR) in a sustained and controlled manner, were prepared and were nontoxic. Most prepared hydrogel DDSs were found to release anti-cancer drugs with first-order or zero-order kinetics. The drug release mechanism was generally denoted as Fickian or non-Fickian transport. The possibility of controlling the kinetics of drug release by changing the pH of the environment was also observed. The findings indicate that these PU hydrogels are suitable for use as intelligent DDSs for the targeted delivery of 5-FU or FUdR. We expect that the hydrogel DDSs developed will be utilized in the treatment of pancreatic cancer.

## 1. Introduction

Cancer remains one of the most significant challenges facing modern medicine, pharmacy, and biomaterials engineering. According to global statistics, approximately one in five people will develop cancer during their lifetime, and approximately one in nine men and one in twelve women will die from it. Estimates suggest that the most common type of cancer is breast cancer, followed by prostate and colon cancer. The most common causes of cancer-related death are lung, colon, and breast cancer [1,2]. Pancreatic cancer ranks ninth among men and tenth among women in terms of cancer incidence.

Adenocarcinoma is the most common malignant pancreatic tumor and varies in its severity. The most common histopathological subtype of pancreatic adenocarcinoma is pancreatic ductal adenocarcinoma (PDAC). The incidence of PDAC is increasing worldwide. This cancer has a high mortality rate due to its aggressive biology and usually delayed diagnosis at a very advanced stage, when metastases have already occurred. Therefore, the pancreatic tumor is mostly inoperable. Treating pancreatic cancer, even without metastases, is difficult. Adjuvant treatment, primarily chemotherapy, is administered for 8–12 weeks after surgery [3].

The most studied drug used for treating pancreatic cancer is 5-Fluorouracil (5-FU). Most trials show a 20% response rate to this chemotherapy agent. The most common treatment regimen is the intravenous administration of 500 mg/m^2^ of body surface area daily for 5 days every 6 months. Local therapies offer hope for improving the prognosis for PDAC patients. However, no commercially available DDSs can currently be administered intratumorally or during endoscopic retrograde cholangiopancreatography to the pancreatic duct [4]. As 5-FU is a water-soluble drug, it is administered intravenously. However, the appearance of drug resistance and low bioavailability is a substantial limitation in the clinical usage of this drug. Moreover, 5-FU results in severe toxicological damage to the gastrointestinal system and blood factors, as well as in neurological, dermatological, and cardiological reactions. Therefore, the development of new polymeric drug delivery systems (DDSs) for 5-FU is essential to achieve a better therapeutic effect with fewer side effects [5,6]. It is worth mentioning that 5-Fluoro-2′-deoxyuridine (FUdR) (fluoropyrimidine), is also used in cancer therapy, primarily for colorectal cancer and other malignancies. It is a 5-FU derivative, and its primary mechanism involves inhibiting thymidylate synthase, a crucial enzyme in DNA synthesis. While 5-FU is more widely used, FUdR has shown the potential for improved efficacy and reduced toxicity in certain contexts, particularly when administered regionally or with specific schedules [7].

One of the most important purposes of the pharmaceutical technology is to discover and engineer new DDSs that contain 5-FU or FUdR, aimed to minimize side effects but also to maximize its clinical efficacy. To date, several 5-FU-containing DDSs (nanoparticles, conjugates, dendrimers) have been developed using the following polymers, among others: chitosan (CHIT), poly(є-caprolactone) (PCL), ethylene oxide and propylene oxide copolymer, polylactide (PLA), poly(ethylene glycol) (PEG) or copolymers of є-caprolactone (CL), *rac*-lactide (LA), and glycolide [5,8,9,10].

A fascinating solution that is increasingly considered in research and development is the use of hydrogel carriers for anti-cancer drugs. Most hydrogels are characterized by good sorption abilities, biocompatibility, relatively high physical and chemical resistance under physiological conditions, similarity to human tissues, sensitivity to environmental conditions, and biodegradability [11,12]. Hydrogel DDSs can be obtained from natural, semi-synthetic, and synthetic polymers [13,14]. Stimulus-responsive hydrogels seem to be particularly useful in cancer chemotherapy. They undergo activation under the influence of various external factors (physical—temperature, magnetic field, and light; chemical—pH, ionic strength, or the presence of specific natural or synthetic chemical compounds, and of enzymes) [15]. In addition to the previously mentioned polymers, peptides and glycopeptides are also used in stimuli-sensitive hydrogel technology. Their three-dimensional network structure endows them with remarkable mechanical resilience, self-healing capacity, and stimuli-responsive behavior, enabling diverse biomedical applications in tissue regeneration, drug delivery, and various therapies. Polyurethanes (PUs) are one of the most biocompatible groups in biomedical polymers used in DDSs technology, particularly those responding to chemical, physical, and biological stimuli [16,17,18]. A pH-responsive PU carrier helps to modulate and improve drug delivery through on-site targeting. Several types of stimuli-responsive PUs have been developed as anti-cancer drug carriers. The resulting PU anti-cancer DDSs responds to the single, dual, or multiple stimuli. The dual-stimulus-responsive PUs with pH responsiveness are pH- and thermo-responsive, pH- and photo-responsive, pH- and shape memory-responsive, and pH- and redox-responsive. Within these dual-responsive PUs, pH- and thermo-responsive PUs and pH- and redox-responsive PUs have been reported for application in anti-cancer DDSs [19,20,21,22,23,24,25]. The main anti-cancer drugs in pH-responsive PU systems are doxorubicin, paclitaxel, and 5-FU. In many cases, the PUs swelled at an acidic pH (the environment of tumor pH in some types of cancer) and demonstrated a controlled drug release. The acid-labile linkages include hydrazone and oxazoline linkages. The hydrolysis of these linkages occurs at a pH range of 4.0 to 6.0. The basic ionizable moieties in pH-responsive PUs include 2-(diethylamino) ethyl methacrylate, 2-(diisopropylamino) ethyl methacrylate, 2-hydroxy ethyl piperazine, diethanolamine, *N*-methyldiethanolamine, and pyridine. These PUs with basic ionizable moieties responded to pH environments ranging from 4.0 to 6.8. On the other hand, the acidic ionizable moieties include 2,2-dimethylol propionic acid, lactic acid, glycolic acid, mercaptoacetic acid, sodium alginate, lysine, arginine, and glutamine. Most of these groups respond to pH environments ranging from 7.4 to 10.4 [19,26,27,28,29,30,31,32,33,34,35,36,37,38,39,40,41,42,43,44,45,46,47,48,49,50,51].

The presented work continues our previous work on hydrogel DDSs containing anti-cancer drugs [52,53,54]. The main goal of this study was to develop PU hydrogels containing 5-FU and FUdR as prospective implantable DDSs for pancreatic cancer therapy (primarily PDAC), distinguished by precise drug delivery and a highly controlled release of these anti-cancer drugs. Firstly, the polyurethane hydrogels with the following structure have been obtained and characterized: hexamethylene diisocyanate (HDI)/CL-LA-PEG/poly(ethylene glycol)-*block*-poly(propylene glycol)-*block*-poly(ethylene glycol) (PEO-b-PPO-b-PEO)/1,4-butanediol (BD)/L-glutamine (Gln) containing 5-FU and FUdR. To the best of our knowledge, there are currently no recognized commercial implantable DDSs of this nature available for the treatment of pancreatic cancer. Furthermore, this category of systems has yet to be distinctly defined or thoroughly discussed.

## 2. Results and Discussion

### 2.1. Synthesis and Characterization of CL, LA, and PEG Copolymers

**Figure 1:**
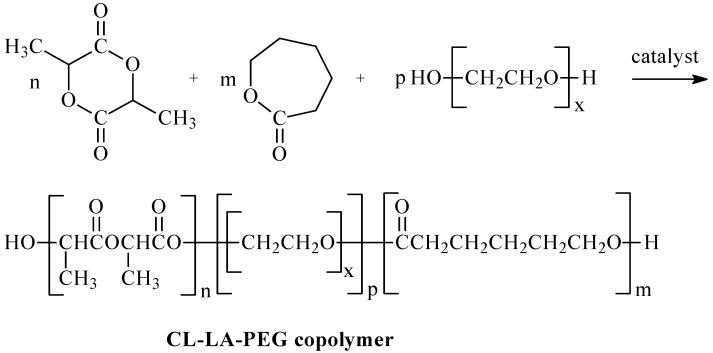
ROP of CL, LA, and PEG in the presence of ZnEt_2_/EDHB catalytic system.

**Figure 2:**
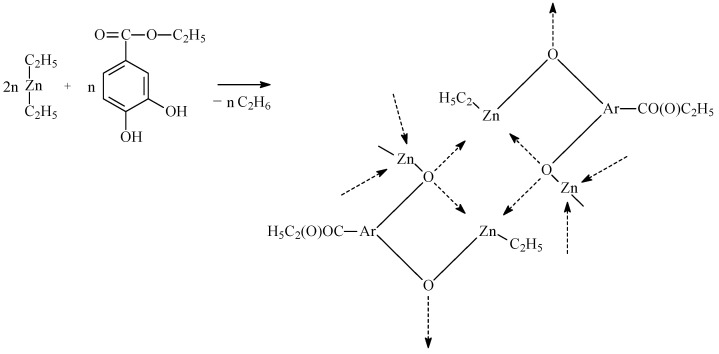
General scheme of synthesizing the catalytic system (ZnEt_2_/EDHB).

The first stage of the work aimed to obtain CL, LA, and PEG copolymers with different microstructures, which were one of the primary raw materials for obtaining hydrogel DDSs containing anti-cancer drugs (Figure 1). The copolymers were synthesized by a ring-opening polymerization process (ROP) in the presence of a new zinc-catalytic system. The catalytic system was obtained in the reaction of diethylzinc (ZnEt_2_) and ethyl-3,4-dihydroxybenzoate (EDHB) in a molar ratio of two to one (Figure 2).

The copolymerization of CL, LA, and PEG was carried out by the bulk method in various molar ratios of the reactants, temperature, and time (according to factorial design—2^3^, with three additional experiments in the center of the design (x_1_ = x_2_ = x_3_ = 0) (Table 1, Table 2 and Table 3).

**Figure 3:**
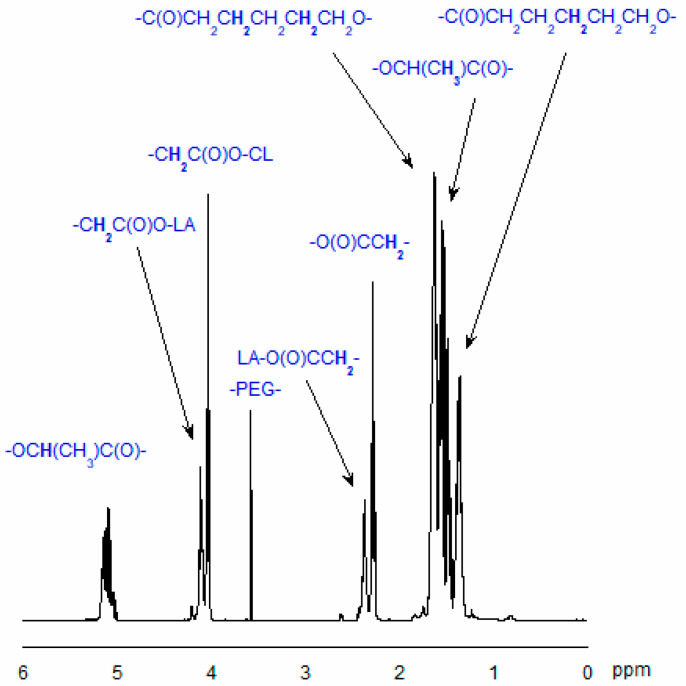
^1^H-NMR spectrum of the CL, LA, and PEG copolymer (in CDCl_3_).

**Figure 4:**
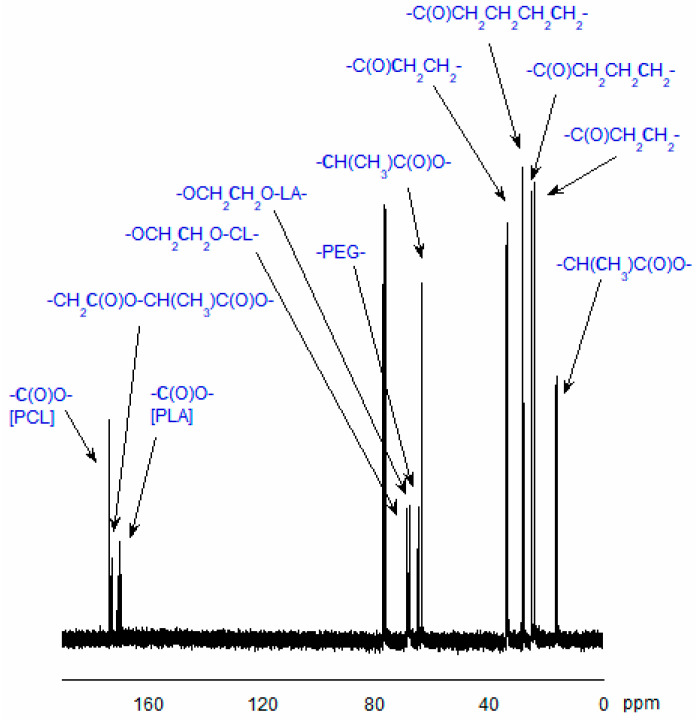
^13^C-NMR spectrum of the CL, LA, and PEG copolymer (in CDCl_3_).

The structure of the obtained copolymers was confirmed by ^1^H- or ^13^C-NMR techniques (see Section 3.7). Figure 3 and Figure 4 show typical spectra of the synthesized CL, LA, and PEG copolymers.

Our primary goal was to obtain the hydrogel DDSs characterized by controlled anti-cancer drug-release kinetics. Our previous experience indicated that one of the main determinants of the polyurethane hydrogel DDSs properties is the microstructure of the soft segments (in this case, copolymers) [55,56]. Therefore, we aimed to obtain copolymers with different CL and LA unit compositions while maintaining similar M_n_, thereby controlling drug release kinetics. To obtain copolymers with significantly different compositions of CL and LA units in the copolymer chain, a copolymerization reaction was carried out in the molar ratios 1:1, 0.6:0.4, and 0.7:0.3 (Table 1, Table 2 and Table 3). Based on the applied mathematical model and the experiments performed, optimal ROP conditions were determined for each of the mentioned comonomer molar ratios.

A full factorial design with three factors at two levels (2^3^) was implemented, and the coefficients of the resulting linear regression models in coded variables were determined using the least squares method.

The polynomial equation for all the individual responses in terms of coded independent factors was as follows:

y_1_ = b_0_ ± b_1_x_1_ ± b_2_x_2_ ± b_3_x_3_

y_2_ = b_0_ ± b_1_x_1_ ± b_2_x_2_ ± b_3_x_3_

Based on the results of three experiments repeated in the center of the design (x_1_ = x_2_ = x_3_ = 0), the following were calculated: repeatability variance (s^2^_rep_), standard deviation (s_rep_), standard deviation of regression coefficients (s_b,i_), t_cal,i_, and regression coefficients (b_i_).

The following results were obtained:

1. Copolymers of CL, LA, and PEG-1500 (molar ratio CL:LA—1:1).
  For process yield:
  y_1_ = 93.2936 + 2.0825x_1_ − 1.3025x_2_ − 6.5625x_3_
  For CL contained in the copolymer chain:
  y_2_ = 0.6882 − 0.0213x_1_ − 0.0813x_2_ + 0.0038x_3_
2. Copolymers of CL, LA, and PEG-1500 (molar ratio CL:LA—0.6:0.4).
  For process yield:
  y_1_ = 95.2255 + 2.3813x_1_ − 1.5288x_2_ − 5.5663x_3_
  For CL contained in the copolymer chain:
  y_2_ = 0.7518 − 0.0213x_1_ − 0.0863x_2_ + 0.0038x_3_
3. Copolymers of CL, LA, and PEG-1500 (molar ratio CL:LA—0.7:0.3).
  For process yield:
  y_1_ = 94.0409 + 2.2525x_1_ − 1.415x_2_ − 6.3475x_3_
  For CL contained in the copolymer chain:
  y_2_ = 0.7891 − 0.0225x_1_ − 0.09x_2_ + 0.005x_3_

Based on the analysis of the results of the 2^3^-factorial design and repetitions in the center of the design for all cases, it was found that there are no grounds to reject the hypothesis about the adequacy of the linear regression equation (F-test), F_cal_ < F_crit._

In order to obtain the expected polymer structure (expected content of individual CL and LA units in the polymer chain) resulting from the reaction stoichiometry, the polyreaction should be carried out under the following conditions: (i) temp.—150 °C, time—36 h for the molar ratio of CL to LA 1:1 (Table 1), (ii) temp.—150 °C, time—36 h for the molar ratio of CL to LA 0.6:0.4 (Table 2), (iii) temp.—150 °C, time—12 h for the CL to LA molar ratio 0.7:0.3 (Table 3). For all reactions, the yield was approximately 100%.

Next, for the established optimal conditions, three copolymers were synthesized, differing in the content of CL, LA, and PEG units in the polymer chains (Table 4). The obtained copolymers were characterized by an average weight molecular mass (*M*_n_) and mol. CL content in the copolymer chain: *M*_n_ = 4300 g/mol (CL = 0.59) for CLLAPEG-4-OC, 4200 g/mol (CL = 0.66) for CLLAPEG-4A-OC, and 4200 g/mol (CL = 0.72) for CLLAPEG-3B-OC, respectively. The *M*_n_ values of the polymers measured by GPC were consistent with the *M*_n_ calculated from ^1^H NMR. Moreover, the dispersity (*Đ*) of the examined polymers was relatively low (approximately 1.4–1.5), indicating a satisfactory level of control over the polymerization process.

### 2.2. Hydrogel DDSs Preparation

**Figure 5:**
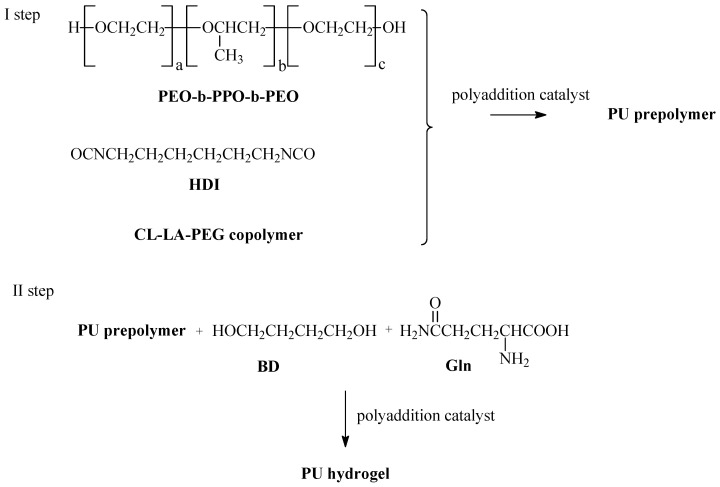
PU hydrogels synthesis.

In the next step, PU hydrogels were obtained from the synthesized CLLAPEG-4-OC, CLLAPEG-4A-OC, and CLLAPEG-3B-OC copolymers (CL-LA-PEG) by the prepolymer method. In the first step, the prepolymers were obtained in the polyaddition process of hexamethylene diisocyanate (HDI), CL-LA-PEG copolymer and poly(ethylene glycol)-*block*-poly(propylene glycol)-*block*-poly(ethylene glycol) (PEO-b-PPO-b-PEO). In the second step, the prepolymers were reacted with 1,4-butanediol (BD) and L-glutamine (Gln). Dibutyltin dilaurate (DBTDL) was used as a catalyst. The molar ratio of the reactants HDI/CL-LA-PEG/PEO-b-PPO-b-PEO/BD/Gln was 3.05:0.75:0.75:0.5:1. The 0.1 wt.% DBDL as a catalyst has been added (Figure 5). HPU-1, HPU-2, and HPU-3 were obtained from the CLLAPEG-4-OC, CLLAPEG-4A-OC, and CLLAPEG-3B-OC copolymers, respectively (Table 5).

The swelling characteristics of the synthesized hydrogels were assessed by measuring the mass swelling ratio (MSR), following a 24 h immersion period (Figure S1). The MSR values recorded were 187%, 229%, and 243% for HPU-3, HPU-2, and HPU-1, respectively. This pattern suggests that the swelling ability of the hydrogels diminishes as the proportion of hydrophobic CL units in the polymer backbone increases, aligning with the anticipated effect of copolymer composition on water absorption.

The complex shear modulus (G*) of the obtained PU hydrogels has been measured.

It was found that there is a relationship between the value of G* and MSR values of the obtained PU hydrogels (refer to Figures S2 and S3). The values of G* were 235 Pa, 595 Pa, and 1950 Pa for HPU-1 (MSR = 243%), HPU-2 (MSR = 229%), and HPU-3 (187%), respectively. It is worth noting that recorded G* coefficient values are within the scope of the dynamic range of mechanical behavior exhibited by various soft and hard human tissues [57,58]. According to the literature data, the G* coefficient is as follows: 300–400 Pa for human brain tissue, 4–12 kPa for the outer layer of human skin (called the stratum corneum), 0.1–2.5 MPa for human articular cartilage, 0.5–1 kPa for the human colon, 5–50 kPa for human liver tissue and 120–1000 Pa for the pancreas. As is known, the mechanical adjustment of implant materials reduces foreign body responses. The results indicate that the DDSs developed in our study may be appropriate for use as implantable biomaterials or coatings.

The cytotoxic studies of the synthesized PU hydrogels have been carried out. It has been found that HPU-1, HPU-2, and HPU-3 showed no significant toxicity to epithelial A549 cells. The mean toxicity was as follows: for HPU-1—90.18 ± 3.05 (200 mg/mL), for HPU-2—92.93 ± 4.36 (190 mg/mL), and for HPU-3—94.76 ± 3.56 (75 mg/mL). The results are presented as a percentage of the control group (which consists of untreated cells cultured concurrently). Toxicity tests were conducted over a duration of 72 h.

Finally, hydrogel DDSs of 5-FU and FUdR were prepared using the incorporation method, in which the drug was physically entrapped within the hydrogel matrix. The drug loading was 5 wt.%, relative to the total weight of the DDSs.

### 2.3. Drug Release and DDS Degradation Studies

**Figure 6:**
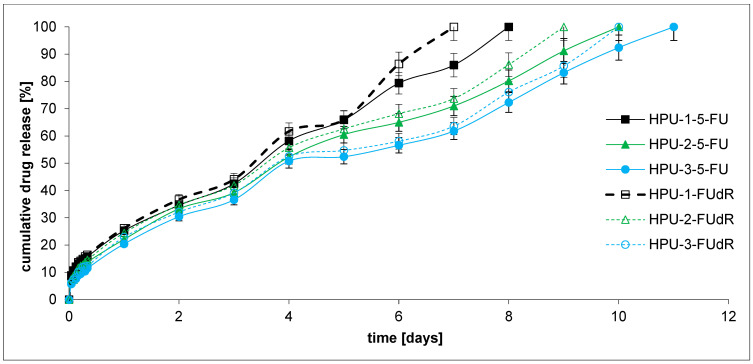
Drug release profiles at pH 7.4.

**Figure 7:**
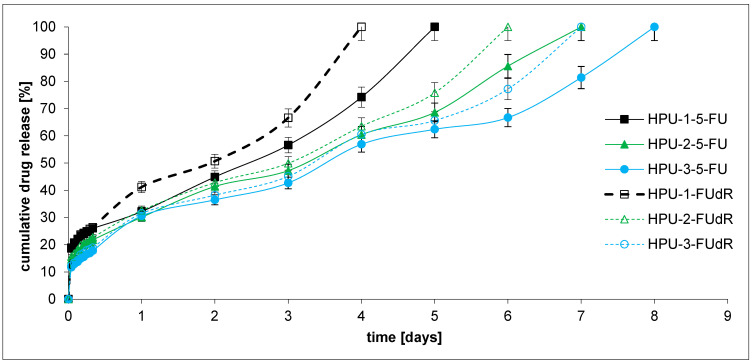
Drug release profiles at pH 8.5.

In the next stage, the in vitro drug release profiles of the obtained pH-sensitive PU hydrogels (DDSs) containing 5-FU or FUdR were investigated to determine their potential as antitumor DDSs. The study’s first aim was to demonstrate how the microstructure of the copolymer CL-LA-PEG chain, which is the main component of hydrogels, affects the kinetics of drug release. The second objective was to analyze the sensitivity of the hydrogel DDSs to the changes in environmental pH, which consequently influences drug release kinetics. We were also keen to explore the rate at which active agents with diverse hydrophilic and hydrophobic traits would be released from the developed hydrogels. As mentioned in the introduction, the intended application goal of our work was to develop hydrogel DDSs dedicated to treating PDAC. Bile, present in this organ’s environment, is alkaline and may affect the release kinetics of the active substance administered topically. Cytostatic drug release tests from hydrogel DDSs were carried out at pH = 7.4 and 8.5 at 37 °C (Figure 6 and Figure 7). The plot’s ordinate was calculated based on the cumulative amount of 5-FU or FUdR released, concerning their initial amount in the hydrogel DDSs.

Within 8–11 days, 5-FU was fully released from the hydrogel PUs at pH 7.4 and 5–8 days at pH 8.5, while FUdR was released within 7–10 days at pH 7.4 and 4–7 days at pH 8.5. The obtained release profiles appear to be satisfactory. Current therapies for pancreatic cancer include 5-FU infusions at a dose of 2400 mg over 46 h. Folinic acid-biomodulated 5-FU is also used. The dose of 5-FU is 425 mg/m^2^ daily for 5 days and every 28 days, or 500 mg/m^2^ every 7 days for 6 weeks [59].

Generally, FUdR was released faster than 5-FU from the obtained hydrogel DDSs at pH 7.4 and 8.5. The result seems logical because FUdR is more soluble in the buffer solution than 5-FU. For example, the percentage of the FUdR released after 7 days of incubation was about 100, 73.7, and 63.6% from the HPU-1-FUdR, HPU-2-FUdR, and HPU-3-FUdR at pH 7.4, respectively. For comparison, 86.0, 71.0, and 61.8% of 5-FU was released from HPU-1-5-FU, HPU-2-5-FU, and HPU-3-5-FU at 7.4, respectively. In the case of the release test conducted at pH 8.5, the same trend was observed as in the experiment at pH 7.4.

The findings imply that the rate of drug release from the hydrogel DDSs decreases as the CL content in the polymer chain increases. For example, 100, 73.7, and 63.6% of the compounds were released from the HPU-1-FUdR (0.59 CL), HPU-2-FUdR (0.66 CL), and HPU-3-FUdR (0.72 CL) at pH 7.4 after 7 days, respectively. An identical relationship was observed for 5-FU release at pH 8.5 and FUdR release at pH 7.4 and 8.5. This is likely due to the increased hydrophobicity of the soft segments composed of copolymers. The system’s hydrophobicity change results from increasing CL units in the polymer chain.

Moreover, the influence of the pH change in the acceptor fluid on the kinetics of anti-cancer drug release was observed. It was observed that the active substances were released faster at pH 8.5 than at pH 7.4. For example, 100% of FUdR was released from HPU-1-FUdR after 7 days at pH 7.4 and 4 days at pH 8.5. The situation was analogous to all DDSs, which contained 5-FU and FUdR. It can be concluded that all obtained DDSs demonstrated pH-sensitive behavior and released drugs at a high rate. This pH response is likely generated by ionized carboxyl groups (Gln), which cause repulsion between the polymer chains and increase swelling. Therefore, the chain structure of the soft segments (CL-LA-PEG copolymers) and the presence of Gln residues in the PU chains can regulate the drug release kinetics from different DDSs. A similar phenomenon was observed by Shoaiba and coworkers, who obtained PUs containing imatinib from HDI, PEG, and various amino acids. Depending on the chain extender used in the PU synthetase (acidic or basic amino acid), a change in the kinetics of the drug release from the carrier was observed [40].

The obtained data for the 5-FU and FUdR release studies were subject to zero-order and first-order kinetics, Korsmeyer–Peppas, and Higuchi models for the evaluation of the kinetics and mechanisms of drug release from hydrogels (Table 6). As is commonly known from the literature, according to the Korsmeyer–Peppas model, for the diffusion–degradation-controlled drug release system, the release exponent value n is in the range of 0.45 and 0.89 (anomalous, non-Fickian). In contrast, when n is close to 0.45, the diffusion (Fickian diffusion) predominates in the process, and, in the opposite case, n > 0.89, the model corresponds to the super case II transport [60].

It was found that the 5-FU and FUdR release kinetics from all DDSs at pH 7.4 followed the near-zero-order model (R^2^ = 0.9806–0.9897). Similarly, 5-FU was released from HPU-1-5-FU, HPU-2-5-FU, and HPU-3-5-FU at pH 8.5 with rather zero-order kinetics (R^2^ = 0.9454–0.9738). On the other hand, it was noted that FUdR was released from the HPU-1-FUdR, HPU-2-FUdR, and HPU-3-FUdR at pH 8.5 with rather first-order kinetics (R^2^ = 0.9466–0.9748).

The analysis of 5-FU and FUdR release from DDSs at pH 8.5 data using the Korsmeyer–Peppas model suggested that all the hydrogels were governed instead by a Fickian transport (n = 0.248–0.377, R^2^ = 0.9339–0.9685). At pH 7.4, drug release from DDSs occurs according to the Fickian (HPU-1-5-FU, HPU-1-FUdR) or non-Fickian mechanism (HPU-2-5-FU, HPU-3-5-FU, HPU-2-FUdR, HPU-3-FUdR). The non-Fickian transport dominates for hydrogels containing a higher concentration of CL units in polymer chains (HPU-2-5-FU (0.66 CL), HPU-3-5-FU (0.72 CL), HPU-2-FUdR (0.66 CL), HPU-3-FUdR (0.72 CL)).

Moreover, according to the 5-FU and FUdR release mechanism considerations, all the examined profiles match the Higuchi model perfectly, implying a diffusional release mechanism (R^2^ = 0.9715–0.9817 at pH 7.4, R^2^ = 0.9252–0.9625 at pH 8.5).

**Figure 8:**
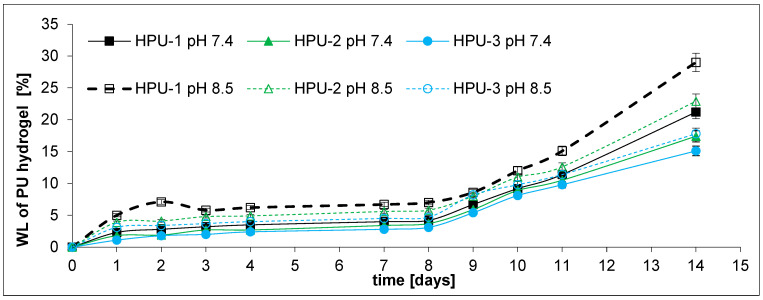
Hydrolytic degradation of PU hydrogels.

Simultaneously with the drug release experiment, hydrolytic degradation studies of the PU hydrogels were carried out. The experiments were conducted at pH 7.4 and 8.5 for 14 days (Figure 8).

Following 4 days of incubation, it was determined that the degradation percentages were between 2.4% and 3.5% at pH 7.4, and between 4.0% and 6.2% at pH 8.5. This implies that the drug’s release rate was markedly higher than the degradation rate. The degradation rates at pH 7.4 were found to be 21.2%, 17.4%, and 15.1% for HPU-1, HPU-2, and HPU-3, respectively, at the end of the 14 day study. At pH 8.5, after 14 days, the weight loss percentages were 29.0%, 22.9%, and 17.8% for HPU-1, HPU-2, and HPU-3, respectively. The results obtained are rational, as the analysis of a mathematical model concerning the release of 5-FU and FUdR indicated a diffusive mechanism at play.

Furthermore, it was found that PU degradation occurs faster at pH 8.5 than at pH 7.4. For example, the degradation rate for HPU-1 was 21.2% and 29.0% at pH 7.4 and 8.5, respectively. The results are consistent with those obtained by other authors studying the biodegradation of poly(ester-urethane)s [61,62].

In our previous work, we successfully prepared biodegradable short-term DDSs containing 5-FU from CL and PEG copolymers. The drug was released over 12 h, with first-order kinetics. Fitting the data to the Higuchi and Korsmeyer–Peppas mathematical models indicated that the anti-cancer drug diffusion mechanisms were dominant [52]. Then, we developed a method for preparing DDSs containing 5-FU from CL/PEG or LA/PEG copolymers and HDI. These DDSs released the drug over 6 to 14 days, with zero- or first-order kinetics that were consistent with a diffusion mechanism. However, we were unable to achieve complete control of the drug release [53]. In turn, Kamaci received long-term PU hydrogels with HDI, LDI, or IPDI, releasing 5-FU over several dozen days [63]. Kamaci also received long-term 5-FU-containing systems. Hydrogel carriers were prepared from HDI, L-lysine ethyl ester diisocyanate, or isophorone diisocyanate and CHIT [64].

In our opinion, we have now managed to obtain DDSs that meet the expectations regarding an anti-cancer drugs-controlled release, assuming the time of complete release of 5-FU and FUdR (4–7 days), and pH-sensitivity of the PU carriers. We hope that developed implantable PU hydrogels demonstrate effective properties as potential DDSs because of their biodegradability and long retention time in the body. However, it is important to note that the in vitro experiment is a simplified model of reality, and the research results cannot be easily translated into the human body.

## 3. Materials and Methods

### 3.1. Materials

є-Caprolactone (2-Oxepanone, CL, 97%, Aldrich, Poznan, Poland), *rac*-lactide (3,6-dimethyl-1,4-dioxane-2,5-dione, *rac*-LA, 96%, Sigma-Aldrich, Poznan, Poland), poly(ethylene glycol) 1500 (PEG 1500, Mn = 1500 g/mol, pure, Sigma-Aldrich, Poznan, Poland), diethylzinc (ZnEt_2_, solution 15 wt. % in toluene, Sigma-Aldrich, Poznan, Poland), dichloromethane (DCM, CH_2_Cl_2_, 99.8%, POCH, Gliwice, Poland), toluene anhydrous (Acros Organics, 99.8%, Extra Dry, Gdansk, Poland), poly(ethylene glycol)-*block*-poly(propylene glycol)-*block*-poly(ethylene glycol) (PEO-b-PPO-b-PEO, M_n_ = 2900 g/mol, PEG, 40 wt. %, Sigma-Aldrich, Poznan, Poland), dibutyltin dilaurate (DBTDL, >96.0%, Sigma-Aldrich, Poznan, Poland), 1,6-diisocyanatohexane (hexamethylene diisocyanate, HDI, 98.0%, Aldrich, Poznan, Poland), ethyl-3,4-dihydroxybenzoate (EDHB, 97%, Aldrich, Poznan, Poland) N,N-dimethylformamide (DMF, anhydrous, 99.8%, Sigma-Aldrich, Poznan, Poland), 1,4-butanediol (BD, 99%, Sigma-Aldrich, Poznan, Poland), L-glutamine ((*S*)-2,5-Diamino-5-oxopentanoic acid, Gln, 99%, Sigma-Aldrich, Poznan, Poland), phosphate-buffered saline (PBS, pH 7.40 ± 0.05, ChemPur, Piekary Slaskie, Poland), tris-HCl buffer, 0.1mol pH 8.5 (ChemPur, Piekary Slaskie, Poland), acetonitrile (ACN, gradient grade for liquid chromatography, Merck, Darmstadt, Germany), trifluoroacetic acid (TFA, 99%, Sigma-Aldrich, Poznan, Poland). All the reagents were used as received.

### 3.2. Synthesis of Catalytic Systems

The catalytic system was prepared in an argon atmosphere at room temperature immediately before each polyreaction. The reactions were carried out in a 100 mL three-necked round-bottomed flask. The glass vessel was equipped with a magnetic stirrer. The flask contained a mixture of ZnEt_2_ (0.02 mol) and EDHB (0.01 mol) in a molar ratio of 2 to 1 and toluene as a solvent (35 mL). First, the appropriate amount of EDHB was placed in a three-necked flask under argon and 35 mL of dry toluene was added. The flask was shielded from light and left on a magnetic stirrer for 24 h. Then, under argon, the appropriate volume of the ZnEt_2_ solution in toluene was added. The flask was shielded from light. The content of the flask was vigorously stirred. The reactions were carried out for about 2 h. The volume of ethane evolved was measured in a gas burette.

### 3.3. Copolymerization of CL, rac-LA, and PEG Procedure

The copolymers were synthesized using the bulk ROP method according to the procedure described earlier, with some modifications [54,65]. The ROPs of CL, LA, and PEG were carried out in a 10 mL glass tube. The required amounts of co-monomers were placed in 10 mL polymerization glass ampoules under argon atmosphere and stirred for 2 h. Next, ZnEt_2_/EDHB catalytic system was added to the reactor under an argon atmosphere (as an anhydrous toluene solution). The reaction vessel was then standing in an oil bath at an appropriate temperature using a thermostat (120, 135, or 150 °C) for the required time (12, 24, or 36 h). Then, the polymerization products were cooled down and dissolved in CH_2_Cl_2_. The obtained solutions were washed with dilute hydrochloric acid (5% aqueous solution) and cold methanol (three times). Next, precipitated polymers were dried at room temperature in a vacuum for 2–3 days. ROPs of CL, LA and PEG on a larger scale were carried out analogously, in ground glass test tubes with a capacity of 30 mL.

#### Design of Experiments

A mathematical model describing the CL, LA, and PEG copolymerization process and its optimization was identified as a part of the experimental work.

To investigate the effect of individual parameters on the process, three variables were selected:

z_1_—polyreaction time (h).
z_2_—polyreaction temperature (°C).
z_3_—CL (LA): PEG molar ratio.

The following output variables were assumed:

y_1_—process yield (%).
y_2_—CL contained in the copolymer chain (mol %).

For the study, the maximum (z_i_ max) and minimum (z_i_ max) values of the natural variables, z_i_, and the coded variables, x_i_ (plane core), were selected so that the possible star points of the rotatable plan (α = 1.682) fit within the allowable range of variability. The natural coordinates of the center point (plane center) and the variable step (∆z_i_) were calculated (Table 7).

A full factorial design, 2^3^, was performed, with three additional experiments in the center of the design (x_1_ = x_2_ = x_3_ = 0). The experimental matrix is presented in Table 8.

### 3.4. Preparation of PU Hydrogels

A 250 mL round-bottom, three-necked flask equipped with a mechanical stirrer, a thermometer, and a dropper was used as a reactor to prepare the PU hydrogels. The reaction was carried out in an argon atmosphere. Before the reaction, all reagents were dried for 2 h at room temperature, under reduced pressure, in an argon atmosphere. PU hydrogels were obtained using the prepolymer method. In the first step, the prepolymers were obtained in the polyaddition process of HDI, CL-LA-PEG copolymer, and PEO-b-PPO-b-PEO. The 0.1 wt.% DBTDL solution in toluene has been added as a catalyst. The prepolymerization was carried out in DMF at 80 °C for about 2 h. In the second step, the prepolymers reacted with BD and Gln. DBTDL was used as a catalyst (in the amount of 0.1 wt%). The second step of the reaction was carried out at 80 °C for 4 h. The final molar ratio of the reactants HDI/CL-LA-PEG/PEO-b-PPO-b-PEO/BD/Gln was 3.05:0.75:0.75:0.5:1. The obtained PU hydrogels were dried at room temperature in a vacuum for 2–3 days.

Squares with sides of 2 cm were cut out of PU hydrogel samples. The mechanical properties of the prepared HPUs were determined using a Kinexus Pro rotational rheometer (Malvern, UK). The selected geometry was a parallel plate system (upper plate diameter 20 mm; lower 60 mm). The measurement temperature was 37 °C, controlled within ±0.1 K. The hydrogels were tested in oscillatory frequency sweeps and oscillatory strain sweeps (Figures S2 and S3). Oscillatory frequency sweeps of 0.01–10 Hz were conducted at a fixed strain amplitude of 0.3% strain (Figure S2). The fixed strain amplitude was chosen to be 0.3%, as G* of all HPUs samples is consistent from 0.1% to about 1% strain (Figure S3). The averages of at least 3 hydrogel replicates were collected for a single data point. The presented results are the mean values collected from at least 3 independent syntheses.

### 3.5. Preparation of Hydrogel DDSs Containing 5-FU or FUdR

Following the procedure below, 5-FU or FUdR was loaded into the hydrogels by physical mixing. The drug was dissolved in DCM and added to the hydrogel sample to suspend the drug in the polymer matrix. The obtained DDSs contain around 5.0% drug (m/m). The mixture was dried in a vacuum at room temperature to obtain a drug-loaded hydrogel film [53,63].

### 3.6. Drug Release Studies

In vitro drug release experiments were performed at 37 °C using complete buffer replacement. Vials containing hydrogel films were filled with 5.0 mL of buffer, sealed, and left at 37 °C for a specific time. Then, the solution was removed for further testing and replaced with fresh PBS. Subsequent samples were obtained at selected intervals [53,63]. The release data points were subjected to zero-order, first-order kinetics, and the Higuchi and Korsmeyer–Peppas models, respectively. Calculations were made based on the formulas mentioned below:

(1) Zero-order: F=kt

(2) $$\mathrm{First}-\mathrm{order}:\;\log F=\log F_{0}-\frac{kt}{2.303}$$

(3) $$\mathrm{Higuchi}\;\mathrm{model}:\;\log F=\log F_{0}-\frac{kt}{2.303}$$

(4) $$\mathrm{Korsmeyer}\text{–}\mathrm{Peppas}\;\mathrm{model}:\;F=kt^{n}\left(F<0.6\right)$$

where

F is the fraction of drug released from the matrix after time t;
F_0_ is the initial amount of drug;
k is a model constant and n is the drug release exponent in the Korsmeyer–Peppas model [60].

### 3.7. NMR Measurements

All the spectra were recorded on an Agilent Technologies 400 MHz (Santa Clara, CA, USA) spectrometer. Chloroform-d (CDCl_3_, Sigma-Aldrich) with TMS internal standard (0.1% *v*/*v*) was used as a solvent.

#### 3.7.1. Analysis of Copolymer Structure

The structure of the obtained copolymers and their *M*_n_ were evaluated using the ^1^H and ^13^C NMR techniques. The Mn of the obtained copolymers chain was estimated using the following formulas [54,66]:

M_n_ = 114n_CL_ + 144n_LA_ + 44n_EG_

n_CL_ = (I_CL_/I_EG_)·[2n·(n_EG_ − 1)]

n_LA_ = (I_LA_/I_EG_)·[2n·(n_EG_ − 1)]

where

I_CL_—the integral intensity of the protons adjacent to the carbon atom of CL units (-CO-**CH_2_**-CH_2_-CH_2_-CH_2_-CH_2_-O-) in the copolymeric chain;
I_LA_—the integral intensity of the protons of LA units (-CO-**CH**(CH_3_)-O-);
I_EG_—the integral intensity of methylene protons of PEG (-**CH_2_**-**CH_2_**-O-), and nEG is the average number of ethylene glycol mers in the PEG molecule.

#### 3.7.2. NMR Data

The ^1^H NMR spectrum of CL, LA, and PEG copolymer (CDCl_3_, ppm): 1.40 (-CO-CH_2_-CH_2_-**CH_2_**-CH_2_-CH_2_-O-), 1.48 (-CO-CH(**CH_3_**)-O-), 1.64 (-CO-CH_2_-**CH_2_**-CH_2_-**CH_2_**-CH_2_-O-), 2.30 (-CO-**CH_2_**-CH_2_-CH_2_-CH_2-_CH_2_-O-) in -CO-CL-**CL**- sequences, 2.38 (-CO**CH_2_**-CH_2_-CH_2_-CH_2_-CH_2_-O-) in -CO-LA-**Cap**- sequences, 3.63 (-**CH_2_**-**CH_2_**-O-), 4.05 (-CO-CH_2_-CH_2_-CH_2_-CH_2_-**CH_2_**-O-) in -CO-**CL**-CL- sequences, 4.13 (-CO-CH_2_-CH_2_-CH_2_-CH_2_-**CH_2_**-O-) in -CO-**CL**-LA sequences, 4.21 (-CH_2_-CH_2_-O-CH_2_-**CH_2_**-OCap-), 4.27 (-CH_2_-CH_2_-O-CH_2_-**CH_2_**-O-LA-), and 5.04 ppm (-CO-**CH**(CH_3_)-O-).

The ^13^C NMR spectrum of CL, LA, and PEG copolymer (CDCl_3_, ppm): 17.02 (-CO-CH(**C**H_3_)-O-), 20.49 (-CO-CH(**C**H_3_)-OH) end groups, 24.62 (-CO-CH_2_-**C**H_2_-CH_2_-CH_2_-CH_2_-O), 25.57 (-CO-CH_2_-CH_2_-**C**H_2_-CH_2_-CH_2_-O-), 28.39 (-CO-CH_2_-CH_2_-CH_2_-**C**H_2_-CH_2_-O-), 32.36 (-CO-CH_2_-CH_2_-**C**H_2_-**C**H_2_-CH_2_-OH) end groups, 34.16 (-CO-**C**H_2_-CH_2_-CH_2_-CH_2_-CH_2_-O-), 62.57 (-CO-CH_2_-CH_2_-CH_2_-CH_2_-**C**H_2_-OH) end groups, 63.49 (-CH_2_-CH_2_-O-CH_2_-**C**H_2_-O-CO-), 64.18 (-CO-CH_2_-CH_2_-CH_2_-CH_2_-**C**H_2_-O), 66.77 (-CO-**C**H(CH_3_)-OH) end groups, 69.21 (-CO-**C**H(CH_3_)-O-) and (-CH_2_-CH_2_-O-**C**H_2_-CH_2_-O-CO-), 70.60 (-**C**H_2_-**C**H_2_-O-), 170.90 ppm (-**C**O-CH(CH_3_)-O-) in lactyl units (L) in -CL-**L**-CL- sequences, and 173.57 (-**C**O-CH_2_-CH_2_-CH_2_-CH_2_-CH_2_-O-).

### 3.8. GPC Measurements

Relative average molecular mass and molecular mass distribution were determined by Gel Permeation Chromatography (GPC). The GPC instrument (GPC Max + TDA 305, Viscotek, Malvern, UK) was equipped with Jordi DVB Mixed Bed columns (one guard and two analytical) at 30 °C in DCM, at a flow rate of 1 mL/min with RI detection and calibration based on narrow PS standards.

### 3.9. HPLC Measurements

Using a Dionex system consisting of a pump type 7580, Jetstream II Plus (WO Industrial Electronics, Vienna, Austria) thermostat, and DAD-detector UVD 340S, 5-FU and FUdR determination conditions were developed. A pre-column Phenomenex C-18 4 mm × 3 mm and column Phenomenex Luna C-18 25 cm × 4.6 mm (particle size 5 µm) were used. The analytical wavelength was 266 nm (5-FU) or 265 nm (FUdR), according to the literature [53,67,68]. In the case of 5-FU analysis, the mobile phase was composed of solvent A (H_2_O + 0.1% TFA) and solvent B (acetonitrile (ACN) + 0.1% TFA). The column temperature was 35 °C, the injection volume was 20 µL, and the flow rate was 1.0 mL/min. The described method was moved to a Hitachi Chromaster system consisting of a pump type 5160, thermostat 5310, DAD-detector UVD 5430, and autosampler model 5260. In case of FUdR, a C18 column (5 mm, 150 mm £4.6 mm, ID) was used. The analysis was performed at a temperature of 30 °C and a flow rate of 1.0 mL/min. The mobile phase was composed of 95% 0.1 M (pH 7.4) phosphate buffer solution–methanol. The methods were validated, and the concentrations of 5-FU and FUdR were determined.

### 3.10. Toxicity Studies

All toxicity studies were performed using human A549 epithelial cells (CRM-CCL-185, ATCC, Washington, DC, USA). The cells were cultured in Minimum Essentials Medium (MEM), supplemented with 10% fetal calf serum, 10 U/mL penicillin, and 100 μg/mL streptomycin (Gibco by Thermo Fisher Scientific, Carlsbad, CA, USA). The A549 cells were seeded in 96-well plates at a density of 5 × 10^3^ cells/well and treated for 72 h with the PU hydrogels.

Cell Proliferation Reagent WST-1 (stable tetrazolium salt, Abcam, Cambridge, UK) assay was used to estimate the viability of A549 cell line after treating the cells with PU hydrogels. After treatment, the cells were incubated with WST-1 (10 μL/well, Sigma Aldrich) for 3 h. The absorbance (570 nm) of the solution was measured in the plate reader (Omega Microplate Spectrophotometer BioTek Instruments, Inc., Winooski, VT, USA).

## 4. Conclusions

This investigation successfully developed PU hydrogels as DDSs for 5-FU and FUdR, which allowed for the sustained and controlled release of these anti-cancer agents. The synthesis of the hydrogels involved hexamethylene diisocyanate, copolymers of CL, LA, and PEG, in addition to PEO-b-PPO-PEO, BD, and Gln. Significantly, the copolymers were produced using a novel and effective catalytic system that resulted from the reaction of ZnEt_2_ with EDHB. The impact of copolymer composition on the kinetics of drug release was thoroughly assessed. It was shown that 5-FU and FUdR were released according to either zero-order or first-order kinetics, predominantly influenced by anomalous (non-Fickian) transport mechanisms. Notably, by adjusting the composition of the soft segments (CLLAPEG) and integrating Gln as a pH-responsive component within the PU chains, the drug release profiles could be precisely tailored over a duration of 5 to 11 days. Moreover, the mechanical and physicochemical properties of the obtained DDSs have promise regarding their potential application as active implantable materials for treating pancreatic cancer.

The DDSs obtained demonstrate pH-sensitive properties and controlled release features that are suitable for localized, sustained chemotherapy. This presents considerable promise for the creation of implantable drug depots intended for the treatment of PDAC—a cancer type known for its unfavorable prognosis, resistance to drugs, and a dense stromal matrix that hinders systemic drug absorption. The capability to administer therapeutically significant doses of 5-FU and FUdR in a controlled manner may lead to improved effectiveness and diminished systemic toxicity.

Further in vivo investigations are required to validate the ability of these DDSs to provide adequate drug concentrations directly to tumor tissues and to assess the therapeutic response and biocompatibility of the system within a PDAC model.

## Figures and Tables

**Table 1:** Copolymers of CL, LA, and PEG-1500 synthesis (molar ratio CL:LA—1:1).

Sample	CL/LA/PEG ^1^	Temp. [°C]	Time [h]	Yield [%]	Yield^cal. 2^ [%]	CL in Polym. ^3^	CL in Polym.^cal. 2^	M_n_ ^4^ [g/mol]
CLLAPEG-1	10:10:1	120	12	99.7	99.0	0.81	0.79	4100
CLLAPEG-2	10:10:1	120	36	98.3	100	0.75	0.74	4200
CLLAPEG-3	10:10:1	150	12	100	96.4	0.59	0.62	4500
CLLAPEG-4	10:10:1	150	36	99.5	100	0.62	0.58	4600
CLLAPEG-5	20:20:1	120	12	87.2	85.9	0.79	0.79	4700
CLLAPEG-6	20:20:1	120	36	91.2	90.1	0.76	0.75	4900
CLLAPEG-7	20:20:1	150	12	76.0	83.3	0.68	0.63	5100
CLLAPEG-8	20:20:1	150	36	90.5	87.5	0.57	0.59	5200
CLLAPEG-9	15:15:1	135	24	92.6	93.2	0.67	0.69	5400
CLLAPEG-10	15:15:1	135	24	94.0	93.2	0.68	0.69	5500
CLLAPEG-11	15:15:1	135	24	96.8	93.2	0.65	0.69	5400

^1^ CL/LA/PEG molar ratio; PEG/catalyst molar ratio—1:0.002; ^2^ calculated from a mathematical model; ^3^ content of CL in the polymer chain—calculated from ^1^H NMR spectra; ^4^ M_n_ calculated from ^1^H NMR spectra.

**Table 2:** Copolymers of CL, LA, and PEG-1500 synthesis (molar ratio CL:LA—0.6:0.4).

Sample	CL/LA/PEG ^1^	Temp. [°C]	Time [h]	Yield [%]	Yield^cal. 2^ [%]	CL in Polym. ^3^	CL in Polym.^cal. 2^	M_n_ ^4^ [g/mol]
CLLAPEG-1A	12:8:1	120	12	100	99.9	0.88	0.86	4000
CLLAPEG-2A	12:8:1	120	36	100	100	0.82	0.81	4200
CLLAPEG-3A	12:8:1	150	12	100	96.8	0.65	0.68	4400
CLLAPEG-4A	12:8:1	150	36	100	100	0.68	0.64	4700
CLLAPEG-5A	24:16:1	120	12	89.8	88.8	0.86	0.86	5700
CLLAPEG-6A	24:16:1	120	36	93.9	93.5	0.83	0.82	5800
CLLAPEG-7A	24:16:1	150	12	78.3	85.7	0.74	0.69	6000
CLLAPEG-8A	24:16:1	150	36	93.2	90.5	0.63	0.65	6100
CLLAPEG-9A	18:12:1	135	24	95.4	95.2	0.73	0.75	5000
CLLAPEG-10A	18:12:1	135	24	96.8	95.2	0.74	0.75	5100
CLLAPEG-11A	18:12:1	135	24	99.7	95.2	0.71	0.75	5000

^1^ CL/LA/PEG molar ratio; PEG/catalyst molar ratio—1:0.002; ^2^ calculated from a mathematical model; ^3^ content of CL in polymer chain—calculated from ^1^H NMR spectra; ^4^ M_n_ calculated from ^1^H NMR spectra.

**Table 3:** Copolymers of CL, LA, and PEG-1500 synthesis (molar ratio CL:LA—0.7:0.3).

Sample	CL/LA/PEG ^1^	Temp. [°C]	Time [h]	Yield [%]	Yield^cal. 2^ [%]	CL in Polym. ^3^	CL in Polym.^cal. 2^	M_n_ ^4^ [g/mol]
CLLAPEG-1B	14:6:1	120	12	100	99.5	0.92	0.90	4100
CLLAPEG-2B	14:6:1	120	36	99.3	100	0.86	0.85	4300
CLLAPEG-3B	14:6:1	150	12	100	96.7	0.68	0.72	4500
CLLAPEG-4B	14:6:1	150	36	100	101.2	0.71	0.67	4800
CLLAPEG-5B	28:12:1	120	12	88.1	86.8	0.90	0.91	5600
CLLAPEG-6B	28:12:1	120	36	92.1	91.3	0.87	0.86	5700
CLLAPEG-7B	28:12:1	150	12	76.8	84.0	0.78	0.73	5900
CLLAPEG-8B	28:12:1	150	36	91.4	88.5	0.66	0.68	6200
CLLAPEG-9B	21:9:1	135	24	97.0	94.0	0.77	0.79	5100
CLLAPEG-10B	21:9:1	135	24	94.5	94.0	0.78	0.79	5000
CLLAPEG-11B	21:9:1	135	24	94.9	94.0	0.75	0.79	5200

^1^ CL/LA/PEG molar ratio; PEG/catalyst molar ratio—1:0.002; ^2^ calculated from a mathematical model; ^3^ content of CL in polymer chain—calculated from ^1^H NMR spectra; ^4^ M_n_ calculated from ^1^H NMR spectra.

**Table 4:** The synthesis of copolymers of CL, LA, and PEG-1500 under optimal conditions.

Sample	CL/LA/PEG ^1^	Temp. [°C]	Time [h]	Yield [%]	CL in Polym. ^2^	M_n_ ^3^ [g/mol]	M_n_ ^4^ [g/mol]	*Đ* ^4^
CLLAPEG-4-OC	10:10:1	150	36	100	0.59	4300	4600	1.45
CLLAPEG-4A-OC	12:8:1	150	36	100	0.66	4200	4300	1.39
CLLAPEG-3B-OC	14:6:1	150	12	100	0.72	4200	4500	1.51

^1^ CL/LA/PEG molar ratio; ^2^ content of CL in polymer chain—calculated from ^1^H NMR spectra; ^3^ M_n_ calculated from ^1^H NMR spectra; ^4^ M_n_ and *Đ* estimated from GPC.

**Table 5:** Characterization of obtained PU hydrogels.

Sample	CL Cont. ^1^	MSR ^2,a^ [%]	MSR ^2,b^ [%]	MSR ^2,c^ [%]	MSR ^2,d^ [%]
HPU-1	0.59	197 ± 9	236 ± 10	241 ± 11	243 ± 9
HPU-2	0.66	182 ± 9	224 ± 9	228 ± 9	229 ± 10
HPU-3	0.72	154 ± 7	182 ± 10	185 ± 9	187 ± 11

^1^ Content of CL in CL/LA/PEG copolymer chain—calculated from ^1^H NMR spectra; ^2^ mass swelling ratio (MSR): a—after 1 h, b—after 2 h, c—after 8 h, d—after 24 h.

**Table 6:** Analysis of anti-cancer drug release results from obtained DDSs using mathematical models.

No.	Zero-Order Model	First-Order Model	Higuchi Model	Korsmeyer–Peppas Model		Transport Mechanism
	*R* ^2^	*R* ^2^	*R* ^2^	*R* ^2^	*n*	
HPU-1-5-FU ^a^	0.9897	0.9602	0.9715	0.9740	0.421	Fickian transport
HPU-2-5-FU ^a^	0.9872	0.9254	0.9817	0.9861	0.477	non-Fickian transport
HPU-3-5-FU ^a^	0.9868	0.8678	0.9737	0.9855	0.499	non-Fickian transport
HPU-1-FUdR ^a^	0.9851	0.9116	0.9587	0.9764	0.424	Fickian transport
HPU-2-FUdR ^a^	0.9845	09635	0.9801	0.9833	0.462	non-Fickian transport
HPU-3-FUdR ^a^	0.9806	0.9359	0.9727	0.9903	0.486	non-Fickian transport
HPU-1-5-FU ^b^	0.9454	0.9394	0.9252	0.9339	0.248	Fickian transport
HPU-2-5-FU ^b^	0.9737	0.9275	0.9580	0.9617	0.329	Fickian transport
HPU-3-5-FU ^b^	0.9738	0.9621	0.9625	0.9679	0.377	Fickian transport
HPU-1-FUdR ^b^	0.9360	0.9466	0.9344	0.9401	0.295	Fickian transport
HPU-2-FUdR ^b^	0.9618	0.9640	0.9463	0.9572	0.308	Fickian transport
HPU-3-FUdR ^b^	0.9715	0.9748	0.9575	0.9685	0.367	Fickian transport

^a^ pH 7.4, ^b^ pH 8.5.

**Table 7:** Plan center and variable steps—copolymers of CL, LA, and PEG-1500 synthesis.

Natural Variable, z_i_	Center of the Plan $z_{i}^{0}=\frac{z_{i}^{max}+z_{i}^{min}}{2}$	Variable Step $∆z_{i}^{0}=\frac{z_{i}^{max}-z_{i}^{min}}{2}$
z_1_	24	12
z_2_	135	15
z_3_	15 or 18 or 21	5 or 6 or 7

**Table 8:** Factorial design (2^3^), experiment matrix, and obtained and calculated results based on the linear model—copolymers of CL, LA, and PEG-1500 synthesis.

Experiment No.	Random Variables		
	x_1_	x_2_	x_3_
1	−1	−1	−1
2	+1	−1	−1
3	−1	+1	−1
4	+1	+1	−1
5	−1	−1	+1
6	+1	−1	+1
7	−1	+1	+1
8	+1	+1	+1
9	0	0	0
10	0	0	0
11	0	0	0

## Data Availability

Data is contained within the article or Supplementary Materials.

## Supplementary Materials

The following supporting information can be downloaded at: https://www.mdpi.com/article/10.3390/ijms262110258/s1.

## Abbreviations

The following abbreviations are used in this manuscript:

BD	1,4-butanediol
CHIT	chitosan
CL	є-caprolactone
CL-LA-PEG	copolymer of є-caprolactone, lactide, and poly(ethylene glycol)
DBTDL	dibutyltin dilaurate
DDSs	drug delivery systems
EDHB	ethyl-3,4-dihydroxybenzoate
5-FU	5-fluorouracil
FUdR	5-fluoro-2′-deoxyuridine
Gln	L-glutamine
HDI	hexamethylene diisocyanate
LA	*rac*-lactide
PCL	poly(є-caprolactone)
PDAC	pancreatic ductal adenocarcinoma
PEG	poly(ethylene glycol)
PEO-bPPO-b-PEO	poly(ethylene glycol)-*block*-poly(propylene glycol)-*block*-poly(ethylene glycol)
PLA	polylactide
PU	polyurethane
PUs	polyurethanes
ZnEt_2_	diethylzinc

## References

[1] Santucci C., Mignozzi S., Levi F., Malvezzi M., Boffetta P., Negri E., La Vecchia C. (2025). European Cancer Mortality Predictions for the Year 2025 with Focus on Breast Cancer. Ann. Oncol..

[2] Bray F., Laversanne M., Sung H., Ferlay J., Siegel R.L., Soerjomataram I., Jemal A. (2024). Global Cancer Statistics 2022: GLOBOCAN Estimates of Incidence and Mortality Worldwide for 36 Cancers in 185 Countries. CA A Cancer J. Clin..

[3] Kwaśniewska D., Fudalej M., Nurzyński P., Badowska-Kozakiewicz A., Czerw A., Cipora E., Sygit K., Bandurska E., Deptała A. (2023). How A Patient with Resectable or Borderline Resectable Pancreatic Cancer Should Be Treated—A Comprehensive Review. Cancers.

[4] Leiphrakpam P.D., Chowdhury S., Zhang M., Bajaj V., Dhir M., Are C. (2025). Trends in the Global Incidence of Pancreatic Cancer and a Brief Review of Its Histologic and Molecular Subtypes. J. Gastrointest. Cancer.

[5] Entezar-Almahdi E., Mohammadi-Samani S., Tayebi L., Farjadian F. (2020). Recent Advances in Designing 5-Fluorouracil Delivery Systems: A Stepping Stone in the Safe Treatment of Colorectal Cancer. Int. J. Nanomed..

[6] Longley D.B., Harkin D.P., Johnston P.G. (2003). 5-Fluorouracil: Mechanisms of Action and Clinical Strategies. Nat. Rev. Cancer.

[7] Gmeiner W.H., Okechukwu C.C. (2023). Review of 5-FU Resistance Mechanisms in Colorectal Cancer: Clinical Significance of Attenuated on-Target Effects. Cancer Drug Resist..

[8] Gu C., Le V., Lang M., Liu J. (2014). Preparation of Polysaccharide Derivates Chitosan-Graft-Poly(ε-Caprolactone) Amphiphilic Copolymer Micelles for 5-Fluorouracil Drug Delivery. Colloids Surf. B Biointerfaces.

[9] Horo H., Das S., Mandal B., Kundu L.M. (2019). Development of a Photoresponsive Chitosan Conjugated Prodrug Nano-Carrier for Controlled Delivery of Antitumor Drug 5-Fluorouracil. Int. J. Biol. Macromol..

[10] Bhadra D., Bhadra S., Jain S., Jain N.K. (2003). A PEGylated Dendritic Nanoparticulate Carrier of Fluorouracil. Int. J. Pharm..

[11] Ullah A., Lim S.I. (2022). Bioinspired Tunable Hydrogels: An Update on Methods of Preparation, Classification, and Biomedical and Therapeutic Applications. Int. J. Pharm..

[12] Kasiński A., Zielińska-Pisklak M., Oledzka E., Sobczak M. (2020). Smart Hydrogels—Synthetic Stimuli-Responsive Antitumor Drug Release Systems. Int. J. Nanomed..

[13] Sobczak M. (2022). Enzyme-Responsive Hydrogels as Potential Drug Delivery Systems—State of Knowledge and Future Prospects. Int. J. Mol. Sci..

[14] Ono K., Hashimoto H., Katayama T., Ueda N., Nagahama K. (2021). Injectable Biocatalytic Nanocomposite Hydrogel Factories for Focal Enzyme-Prodrug Cancer Therapy. Biomacromolecules.

[15] Kesharwani P., Bisht A., Alexander A., Dave V., Sharma S. (2021). Biomedical Applications of Hydrogels in Drug Delivery System: An Update. J. Drug Deliv. Sci. Technol..

[16] Binaymotlagh R., Chronopoulou L., Haghighi F.H., Fratoddi I., Palocci C. (2022). Peptide-Based Hydrogels: New Materials for Biosensing and Biomedical Applications. Materials.

[17] Hasallari F., Gallo E., Rizzuti S., Diaferia C., Salvatore M., Accardo A., Gianolio E., Aime S. (2025). A Biocompatible, Highly Sensitive Hydrogel-Based T1 Thermometer for in Vivo MRI Applications. Mater. Today Chem..

[18] Zhang J., Zou J., Ren J. (2025). Recent Advances in Glycopeptide Hydrogels: Design, Biological Functions, and Biomedical Applications. Front. Bioeng. Biotechnol..

[19] Tan R.Y.H., Lee C.S., Pichika M.R., Cheng S.F., Lam K.Y. (2022). PH Responsive Polyurethane for the Advancement of Biomedical and Drug Delivery. Polymers.

[20] Boffito M., Torchio A., Tonda-Turo C., Laurano R., Gisbert-Garzarán M., Berkmann J.C., Cassino C., Manzano M., Duda G.N., Vallet-Regí M. (2020). Hybrid Injectable Sol-Gel Systems Based on Thermo-Sensitive Polyurethane Hydrogels Carrying pH-Sensitive Mesoporous Silica Nanoparticles for the Controlled and Triggered Release of Therapeutic Agents. Front. Bioeng. Biotechnol..

[21] Iyer R., Nguyen T., Padanilam D., Xu C., Saha D., Nguyen K.T., Hong Y. (2020). Glutathione-Responsive Biodegradable Polyurethane Nanoparticles for Lung Cancer Treatment. J. Control. Release.

[22] Yu C., Tan X., Xu Z., Zhu G., Teng W., Zhao Q., Liang Z., Wu Z., Xiong D. (2020). Smart Drug Carrier Based on Polyurethane Material for Enhanced and Controlled DOX Release Triggered by Redox Stimulus. React. Funct. Polym..

[23] Zhao L., Liu C., Qiao Z., Yao Y., Luo J. (2018). Reduction Responsive and Surface Charge Switchable Polyurethane Micelles with Acid Cleavable Crosslinks for Intracellular Drug Delivery. RSC Adv..

[24] Sun J., Rust T., Kuckling D. (2019). Light—Responsive Serinol—Based Polyurethane Nanocarrier for Controlled Drug Release. Macromol. Rapid Commun..

[25] Aluri R., Saxena S., Joshi D.C., Jayakannan M. (2018). Multistimuli-Responsive Amphiphilic Poly(Ester-Urethane) Nanoassemblies Based on L-Tyrosine for Intracellular Drug Delivery to Cancer Cells. Biomacromolecules.

[26] Zhou L., Yu L., Ding M., Li J., Tan H., Wang Z., Fu Q. (2011). Synthesis and Characterization of pH-Sensitive Biodegradable Polyurethane for Potential Drug Delivery Applications. Macromolecules.

[27] Zhou L., Liang D., He X., Li J., Tan H., Li J., Fu Q., Gu Q. (2012). The Degradation and Biocompatibility of pH-Sensitive Biodegradable Polyurethanes for Intracellular Multifunctional Antitumor Drug Delivery. Biomaterials.

[28] Song N., Zhou L., Li J., Pan Z., He X., Tan H., Wan X., Li J., Ran R., Fu Q. (2016). Inspired by Nonenveloped Viruses Escaping from Endo-Lysosomes: A pH-Sensitive Polyurethane Micelle for Effective Intracellular Trafficking. Nanoscale.

[29] Pardini F.M., Amalvy J.I. (2014). Synthesis and Swelling Behavior of pH-Responsive Polyurethane/Poly[2-(Diethylamino)Ethyl Methacrylate] Hybrid Materials. J. Appl. Polym. Sci..

[30] Yao Y., Xu D., Liu C., Guan Y., Zhang J., Su Y., Zhao L., Meng F., Luo J. (2016). Biodegradable pH-Sensitive Polyurethane Micelles with Different Polyethylene Glycol (PEG) Locations for Anti-Cancer Drug Carrier Applications. RSC Adv..

[31] Kim S., Traore Y.L., Ho E.A., Shafiq M., Kim S.H., Liu S. (2018). Design and Development of pH-Responsive Polyurethane Membranes for Intravaginal Release of Nanomedicines. Acta Biomater..

[32] He W., Zheng X., Zhao Q., Duan L., Lv Q., Gao G.H., Yu S. (2016). pH—Triggered Charge—Reversal Polyurethane Micelles for Controlled Release of Doxorubicin. Macromol. Biosci..

[33] Kim S., Traore Y.L., Chen Y., Ho E.A., Liu S. (2018). Switchable On-Demand Release of a Nanocarrier from a Segmented Reservoir Type Intravaginal Ring Filled with a pH-Responsive Supramolecular Polyurethane Hydrogel. ACS Appl. Bio Mater..

[34] Duru Kamacı U., Kamacı M. (2020). Preparation of Polyvinyl Alcohol, Chitosan and Polyurethane-Based pH-Sensitive and Biodegradable Hydrogels for Controlled Drug Release Applications. Int. J. Polym. Mater. Polym. Biomater..

[35] Bhattacharyya A., Mukhopadhyay P., Kundu P.P. (2014). Synthesis of a Novel pH-sensitive Polyurethane–Alginate Blend with Poly(Ethylene Terephthalate) Waste for the Oral Delivery of Protein. J. Appl. Polym. Sci..

[36] Bhattacharyya A., Mukhopadhyay P., Pramanik N., Kundu P.P. (2016). Effect of Polyethylene Glycol on Bis(2-hydroxyethyl) terephthalate—Based Polyurethane/Alginate pH-Sensitive Blend for Oral Protein Delivery. Adv. Polym. Technol..

[37] Solanki A., Thakore S. (2015). Cellulose Crosslinked pH-Responsive Polyurethanes for Drug Delivery: α-Hydroxy Acids as Drug Release Modifiers. Int. J. Biol. Macromol..

[38] Nabid M.R., Omrani I. (2016). Facile Preparation of pH-Responsive Polyurethane Nanocarrier for Oral Delivery. Mater. Sci. Eng. C.

[39] Hua D., Liu Z., Wang F., Gao B., Chen F., Zhang Q., Xiong R., Han J., Samal S.K., De Smedt S.C. (2016). pH Responsive Polyurethane (Core) and Cellulose Acetate Phthalate (Shell) Electrospun Fibers for Intravaginal Drug Delivery. Carbohydr. Polym..

[40] Shoaib M., Bahadur A., Saeed A., Rahman M.S.U., Naseer M.M. (2018). Biocompatible, pH-Responsive, and Biodegradable Polyurethanes as Smart Anti-Cancer Drug Delivery Carriers. React. Funct. Polym..

[41] Yang H.Y., Zhang X.M., Duan L.J., Zhang M.Y., Gao G.H., Zhang H.X. (2013). Environmental pH-responsive Fluorescent PEG-polyurethane for Potential Optical Imaging. J. Appl. Polym. Sci..

[42] Zhou H., Xun R., Liu Q., Fan H., Liu Y. (2014). Preparation of Thermal and pH Dually Sensitive Polyurethane Membranes and Their Properties. J. Macromol. Sci. Part B.

[43] Wang A., Gao H., Sun Y., Sun Y., Yang Y.-W., Wu G., Wang Y., Fan Y., Ma J. (2013). Temperature- and pH-Responsive Nanoparticles of Biocompatible Polyurethanes for Doxorubicin Delivery. Int. J. Pharm..

[44] Pardini F.M., Faccia P.A., Pardini O.R., Amalvy J.I. (2018). Thermal and pH Dual Responsive Polyurethane/2-(Diisopropylamino)Ethyl Methacrylate Hybrids: Synthesis, Characterization, and Swelling Behavior. Int. J. Polym. Anal. Charact..

[45] Li Y., Chen H., Liu D., Wang W., Liu Y., Zhou S. (2015). pH-Responsive Shape Memory Poly(Ethylene Glycol)–Poly(ε-Caprolactone)-Based Polyurethane/Cellulose Nanocrystals Nanocomposite. ACS Appl. Mater. Interfaces.

[46] Li J., Zhang X., Gooch J., Sun W., Wang H., Wang K. (2015). Photo- and pH-Sensitive Azo-Containing Cationic Waterborne Polyurethane. Polym. Bull..

[47] Guan Y., Su Y., Zhao L., Meng F., Wang Q., Yao Y., Luo J. (2017). Biodegradable Polyurethane Micelles with pH and Reduction Responsive Properties for Intracellular Drug Delivery. Mater. Sci. Eng. C.

[48] Bu L., Zhang H., Xu K., Du B., Zhu C., Li Y. (2019). pH and Reduction Dual-Responsive Micelles Based on Novel Polyurethanes with Detachable Poly(2-Ethyl-2-Oxazoline) Shell for Controlled Release of Doxorubicin. Drug Deliv..

[49] Yu S., He C., Ding J., Cheng Y., Song W., Zhuang X., Chen X. (2013). pH and Reduction Dual Responsive Polyurethane Triblock Copolymers for Efficient Intracellular Drug Delivery. Soft Matter.

[50] Yu S., He C., Lv Q., Sun H., Chen X. (2014). pH and Reduction Dual Responsive Cross-Linked Polyurethane Micelles as an Intracellular Drug Delivery System. RSC Adv..

[51] Song Q., Chen H., Zhou S., Zhao K., Wang B., Hu P. (2016). Thermo- and pH-Sensitive Shape Memory Polyurethane Containing Carboxyl Groups. Polym. Chem..

[52] Kasiński A., Zielińska-Pisklak M., Kowalczyk S., Plichta A., Zgadzaj A., Oledzka E., Sobczak M. (2021). Synthesis and Characterization of New Biodegradable Injectable Thermosensitive Smart Hydrogels for 5-Fluorouracil Delivery. Int. J. Mol. Sci..

[53] Kasiński A., Zielińska-Pisklak M., Oledzka E., Nałęcz-Jawecki G., Drobniewska A., Sobczak M. (2020). Hydrogels Based on Poly(Ether-Ester)s as Highly Controlled 5-Fluorouracil Delivery Systems—Synthesis and Characterization. Materials.

[54] Kasiński A., Świerczek A., Zielińska-Pisklak M., Kowalczyk S., Plichta A., Zgadzaj A., Oledzka E., Sobczak M. (2023). Dual-Stimuli-Sensitive Smart Hydrogels Containing Magnetic Nanoparticles as Antitumor Local Drug Delivery Systems—Synthesis and Characterization. Int. J. Mol. Sci..

[55] Zagórska-Dziok M., Kleczkowska P., Olędzka E., Figat R., Sobczak M. (2021). Poly(Chitosan-Ester-Ether-Urethane) Hydrogels as Highly Controlled Genistein Release Systems. Int. J. Mol. Sci..

[56] Sobczak M., Oledzka E., Kwietniewska M., Nałęcz-Jawecki G., Kołodziejski W. (2014). Promising Macromolecular Conjugates of Camptothecin—The Synthesis, Characterization and in Vitro Studies. J. Macromol. Sci. Part A.

[57] Tabet A., Mommer S., Vigil J.A., Hallou C., Bulstrode H., Scherman O.A. (2019). Mechanical Characterization of Human Brain Tissue and Soft Dynamic Gels Exhibiting Electromechanical Neuro-Mimicry. Adv. Healthc. Mater..

[58] Speidel A.T., Chivers P.R.A., Wood C.S., Roberts D.A., Correia I.P., Caravaca A.S., Chan Y.K.V., Hansel C.S., Heimgärtner J., Müller E. (2022). Tailored Biocompatible Polyurethane-Poly(Ethylene Glycol) Hydrogels as a Versatile Nonfouling Biomaterial. Adv. Healthc. Mater..

[59] Sethy C., Kundu C.N. (2021). 5-Fluorouracil (5-FU) Resistance and the New Strategy to Enhance the Sensitivity against Cancer: Implication of DNA Repair Inhibition. Biomed. Pharmacother..

[60] Dash S., Murthy P.N., Nath L., Chowdhury P. (2010). Kinetic Modeling on Drug Release from Controlled Drug Delivery Systems. Acta Pol. Pharm..

[61] Marín R., Muñoz-Guerra S. (2009). Carbohydrate-Based Poly(Ester-Urethane)s: A Comparative Study Regarding Cyclic Alditols Extenders and Polymerization Procedures. J. Appl. Polym. Sci..

[62] Asefnejad A., Khorasani M.T., Behnamghader A., Farsadzadeh B., Bonakdar S. (2011). Manufacturing of Biodegradable Polyurethane Scaffolds Based on Polycaprolactone Using a Phase Separation Method: Physical Properties and in vitro Assay. Int. J. Nanomed..

[63] Kamaci M. (2020). Polyurethane-Based Hydrogels for Controlled Drug Delivery Applications. Eur. Polym. J..

[64] Kamaci M., Kaya I. (2023). Chitosan Based Hybrid Hydrogels for Drug Delivery: Preparation, Biodegradation, Thermal, and Mechanical Properties. Polym. Adv. Technol..

[65] Domańska I.M., Zalewska A., Cieśla K., Plichta A., Głuszewski W., Łyczko M., Kowalczyk S., Oledzka E., Sobczak M. (2023). The Influence of Electron Beam and Gamma Irradiation on Paclitaxel-Loaded Nanoparticles of Fully Randomized Copolymers in Relation to Potential Sterilization. J. Drug Deliv. Sci. Technol..

[66] Jiang Z., Deng X., Hao J. (2007). Thermogelling Hydrogels of Poly(ε-caprolactone-*co*-D,L-lactide)–Poly(Ethylene Glycol)–Poly(ε-caprolactone-*co*-D,L-lactide) and Poly(ε-caprolactone-*co*-L-lactide)–Poly(Ethylene Glycol)–Poly(ε-caprolactone-*co*-L-lactide) Aqueous Solutions. J. Polym. Sci. A Polym. Chem..

[67] Maring J.G., Schouten L., Greijdanus B., De Vries E.G.E., Uges D.R.A. (2005). A Simple and Sensitive Fully Validated HPLC-UV Method for the Determination of 5-Fluorouracil and Its Metabolite 5,6-Dihydrofluorouracil in Plasma. Ther. Drug Monit..

[68] Wang J.-X., Sun X., Zhang Z.-R. (2002). Enhanced Brain Targeting by Synthesis of 3′,5′-Dioctanoyl-5-Fluoro-2′-Deoxyuridine and Incorporation into Solid Lipid Nanoparticles. Eur. J. Pharm. Biopharm..

