# Supplementary material for: Preparation and Characterization of New pH-Sensitive Polyurethane Hydrogels as Anti-Cancer Drug Delivery Systems for 5-Fluorouracyl and Fluorodeoxyuridine
Source: Int J Mol Sci. 2025 Oct 22;26(21):10258. doi: 10.3390/ijms262110258 (PMC12609683; doi:10.3390/ijms262110258)
Supplement: Supplementary file 1 [file ijms-26-10258-s001.zip › ijms-3909047-supplementary.pdf]

Preparation and characterization of new pH-sensitive polyurethane hydrogels as anti-cancer drug delivery systems for -fluorouracyl and fluorodeoxyuridine

Marcin Sobczak[1,*], Adam Kasiński[1], Karolina Kędra[2], Joachim Frankowski[1], Matylda Kurządkowska[1], Karolina Watrakiewicz[1], Karolina Mulas[1], Katarzyna Strzelecka[1], Marcin Chodkowski[3], Małgorzata Krzyżowska[3], Andrzej Deptała[4,5], Ewa Oledzka[1]

[1] Department of Pharmaceutical Chemistry and Biomaterials, Faculty of Pharmacy, Medical University of Warsaw, Banacha 1 Str., 02-097 Warsaw, Poland; marcin.sobczak@wum.edu.pl (M.S.); adam.kasinski@wum.edu.pl (A.K.); joachim.frankowski@wum.edu.pl (J.F.); matylda.kurzatkowska@wum.edu.pl (M.K.), karolka2804@interia.pl (K.W.), karolina.mulas@wum.edu.pl (K.M.); katarzyna.strzelecka@wum.edu.pl (K.S.); eoledzka@wum.edu.pl (E.O.);

[2] Institute of Physical Chemistry, Polish Academy of Sciences, Kasprzaka 44/52 Str., 01-224 Warsaw, Poland; kkedra@ichf.edu.pl (K.K.)

[3] Division of Medical and Environmental Microbiology, Military Institute of Hygiene and Epidemiology, 01-063 Warsaw, Poland; malgorzata.krzyzowska@wum.edu.pl (M.K.); marcin.chodkowski@wihe.pl (M.Ch.);

[4] Department of Oncology, National Medical Institute of the Ministry of the Interior and Administration, 02-507 Warsaw, Poland; andrzej.deptala@wum.edu.pl (A.D.);

[5] Department of Oncology Propaedeutics, Medical University of Warsaw, 01-445 Warsaw, Poland; andrzej.deptala@wum.edu.pl (A.D.);

***** Correspondence: marcin.sobczak@wum.edu.pl

Supporting information

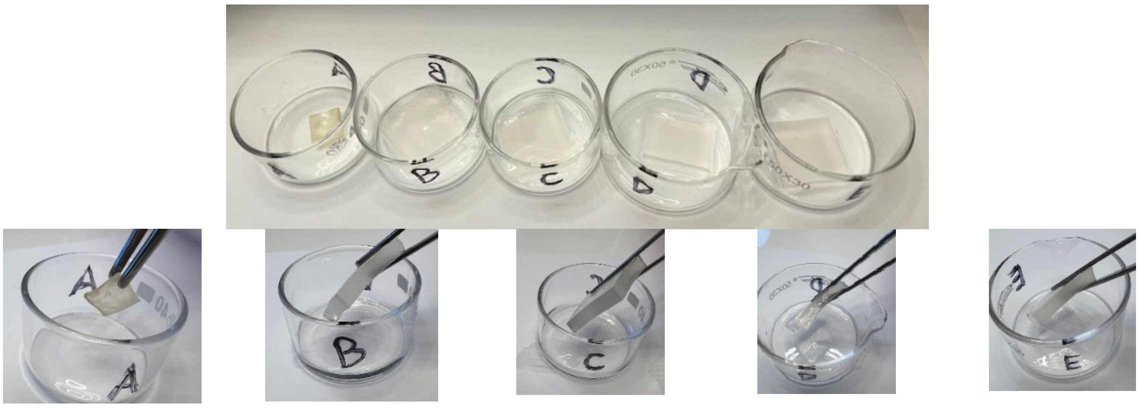

Figure S1. Example photos of the hydrogel (HPU-1) (A) before, (B) after 1h, (C) after 2h, (D) after 8h, and (E) after 24 h the process of buffer-absorbing (buffer solution pH = 7.4).

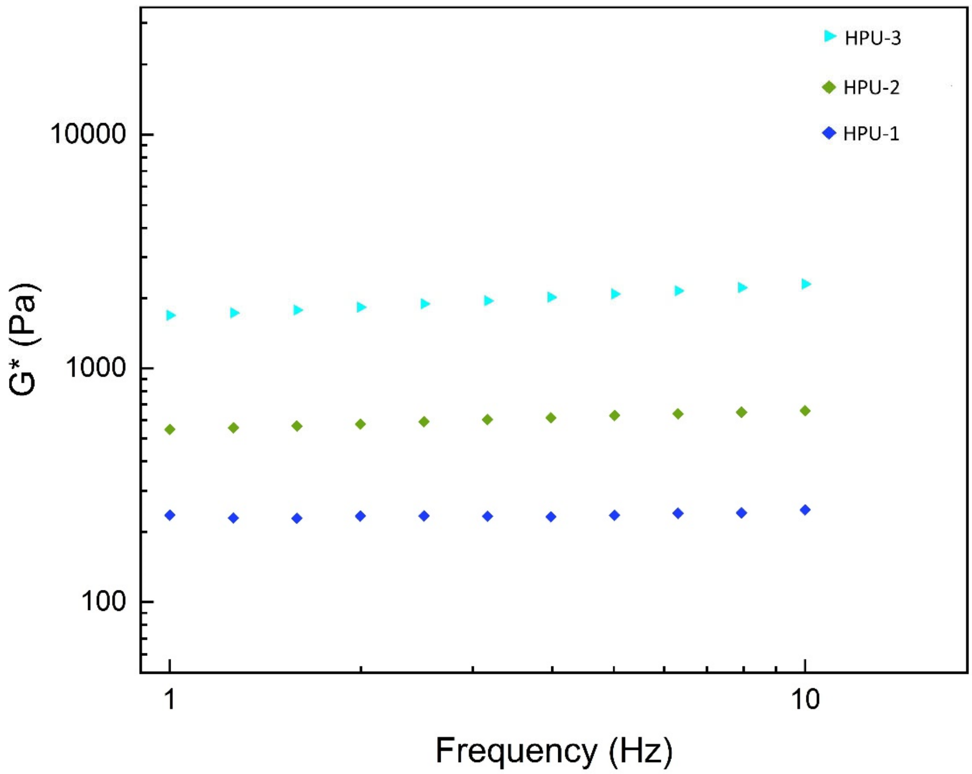

Figure S2. The oscillatory frequency sweeps at fixed strain (0.2 %): complex shear modulus G* of representative HPU samples measured at 37 ℃.

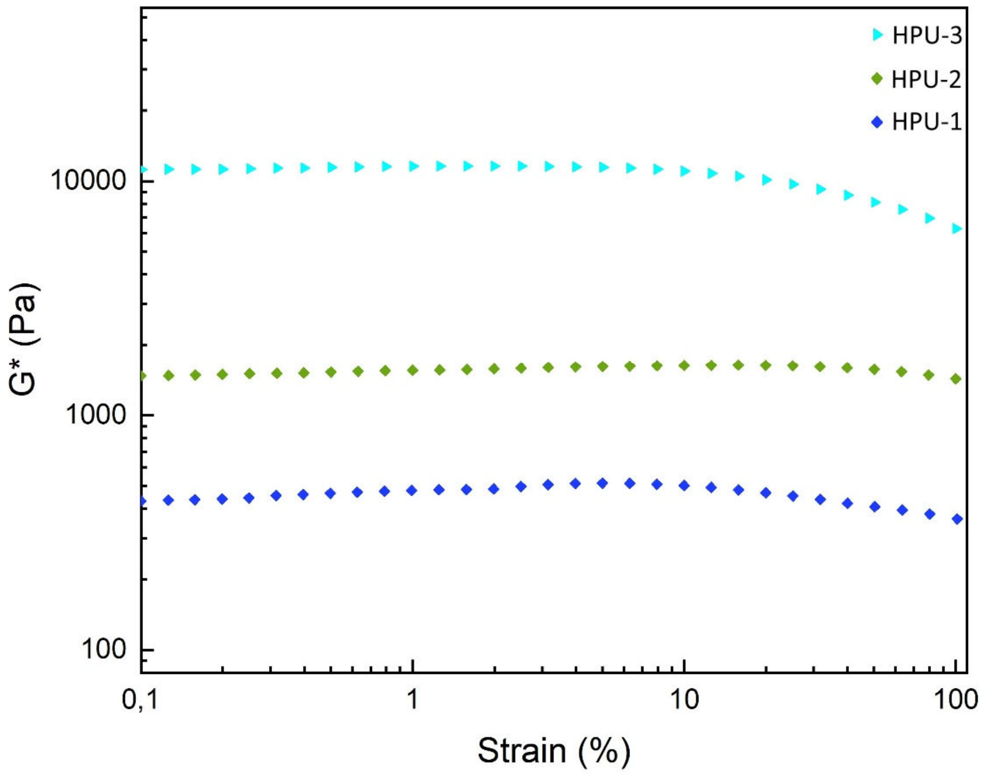

Figure S3. The representative oscillatory strain sweeps, for 0.1 – 100 % strain, at fixed frequency 1.6 Hz, under 37 ℃. Complex shear modulus for the representative HPU samples is consistent from 0.1% to about 1% strain.
